# Traditional-medical knowledge and perception of pangolins (*manis sps*) among the awori people, Southwestern Nigeria

**DOI:** 10.1186/1746-4269-7-25

**Published:** 2011-09-01

**Authors:** Durojaye A Soewu, Temilolu A Adekanola

**Affiliations:** 1 Department of Biological Sciences, Covenant University, P.M.B 1023, Ota, Ogun State, Nigeria; 2 Department of Plant Science and Applied Zoology, Olabisi Onabanjo University, Ago- Iwoye Ogun State, Nigeria

## Abstract

**Background:**

Animals have been used as medicinal resources throughout human history. Majority of wildlife used in traditional medicines is taken from the wild; hence demand by traditional medicine is a cause of over-exploitation of wild animals. Indiscriminate use of endangered species portends grievous implications for biodiversity conservation. This study investigated the dynamics of the use of pangolin in trado-medicinal preparations amongst the Awori people.

**Methods:**

Forty traditional Yorubic-medical practitioners (tymps) selected through stratified random-sampling technique were interviewed using open-ended questionnaires. Various aspects of the utilisation of pangolin in traditional medicinal practices were investigated. Data collected were analysed using simple frequencies and percentages.

**Results:**

An average of 1.6 pangolins were utilised per tymp per month. About 43% of respondents contracted hunters for deliberate searches for the animals. More than 92% believed that pangolins' abundance is steadily decreasing. Above 97% reported a continuous decline in the size of pangolin. Pangolin was used in treating 47 conditions. Situations accommodated included those that can be treated by orthodox medicine like rheumatism and venereal diseases as well as some that are out of range for orthodox medicine including kleptomania and good luck charms. Some substitute animals like gorilla are under a greater conservation threat than pangolin.

**Conclusions:**

Utilisation of pangolin in traditional medicine has no consideration for sustainability. Awareness should be created on people as regards the implications of unsustainable depletion of medicinal resources. Efforts should be intensified on ex-situ breeding of pangolin while subjecting the scales and other parts to laboratory studies to determine the bioactive constituents.

## Introduction

Throughout human history, and in practically every human culture which presents a structured medical system, animals have been used as medicinal resources for the treatment and relieve of a wide variety of human health challenges [1]. Some animals have also been used for religious and cultural purposes such as sacrifices for appeasing and invoking spirits and gods while some others have played important roles in magic rituals and mysticism [2-4]. Traditional medicine has been described by the World Health Organization (WHO) as one of the surest means to achieve total health care coverage for the world's population [5]. The World Health Organisation (WHO) stated that traditional medicine refers to health practices, approaches, knowledge and beliefs incorporating animal and mineral based medicines, spiritual therapies, manual techniques and exercises, applied singularly or in combination to treat, diagnose and prevent illnesses or maintain well-being [6,7]. Traditional medicine was further defined by WHO as the sum total of all knowledge and practices, whether explicable or not, used in diagnosis, prevention and elimination of physical, mental or social imbalance and relying exclusively on practical experiences and observations handed from generation to generation, whether verbally or in writing [7,8]. This practice of treating human diseases by use of therapeutics obtained or ultimately derived from animals is called Zootherapy [9]. Zootherapy on the other hand is an important component of ethnozoology, which deals with the study of relationship between the human societies and the animal resources around them [10]. In modern societies, zootherapy constitutes an important alternative among many other known therapies practiced worldwide [11].

It was observed that many animal species have been over-exploited as sources of medicines for the folk medicine trade [12]. A vast majority of wildlife products used in traditional medicines is usually taken directly from the wild [13]. The demand created by traditional medicine has however been identified as one of the causes of the overexploitation of the wild population of numerous animal species. This indiscriminate use of wild animals, especially endangered species in all forms of traditional medicine is a cause of growing concern [11,12]. Poaching animals for their medicinal values has brought many of the wild species closer to extinction and necessitated their listing in the red data book [14]. It is thus evident that the soaring demand for their body parts for use in medicinal practices is one common dilemma facing all fauna species [15]. Obviously, continued depletion of medicinal wildlife resources not only embodies a challenge for conservation, but more importantly represents a serious threat to the health status of human population [4,13].

In Africa, reliance on wildlife-based medicine stems partly on the one hand from the high cost of conventional medicine and the inaccessibility of modern health care facilities. It is also due on the other hand to the fact that traditional medicine is often deemed a more appropriate method of treatment [13]. Traditional African Medicine (TAM) is a holistic discipline involving extensive use of indigenous herbalism combined with some aspects of African spirituality [16]. A considerable number of people living in rural areas in Africa rely solely on traditional medicines for health care [4,13]. The basis for traditional medicines and the primary ingredients used by the traditional healers are wild animal and plant species. This practice is widespread in Africa, and market stalls selling plants and animal parts for medicines are common in both rural and urban markets in many African towns and cities [17]. Several authors have also recorded a wide variety of animals and their parts in sales for other parts of the world. In a study on animal based remedies in the semi-arid region of Northeastern Brazil, Alves *et al *(2011) [14] reported 51 medicinal animals distributed among 42 zoological families used to cure about 68 ailments. Twenty-four animal species used in 35 different medicinal purposes were documented in an ethnozoological study in Mount Abu wildlife sanctuary in India [10]. In a review, Mahawar and Jaroli (2008) [18] identified 109 animal species with 270 uses in traditional medicine in different parts of India, while Ferreira *et al *(2009) [19] in another study in Crato and Juazeiro do Norte, Ceara, Brazil, recorded 31 animal species distributed among 21 families. A study in some markets in Isreal recorded 20 animal species which products were sold as traditional remedies [10].

There is no indication that the level of utilisation of medicinal wildlife resources for traditional medicine would diminish [13]. On the contrary, there is every reason to believe that the quantities of animals (and plants) required for traditional medicine would increase substantially in years to come as human population grow and acceptance of traditional medicine and natural products increases in the market. Moreso, the magic, superstition and dogma that surrounded traditional medicinal preparations are giving way to an understanding of the real basis of their curative power and consequently their social acceptance [4,13]. According to Alves *et al *(2011) [14], the use of animals for medicinal purposes is part of a body of traditional knowledge which is increasingly becoming more relevant to discussions on conservation biology, public health policies, sustainable management of natural resources, biological prospection and patents. This utilisation of animals in zootherapeutic practices has little or no consideration for the conservation status of the faunistic resources as protected animals are also used indiscriminately [15,20]. More than sixty-six percent of the animal species utilised in zootherapy by the Garasiya people of Rajasthan in India are included in the IUCN Red Data List [10].

Regarding its global conservation status, pangolins are presently rated as *near threatened *on IUCN *Red Data Book *and listed in appendix II of CITES. All four African species are listed in Class B of the 1986 African Convention on Nature and Natural Resources while the three western African species of pangolins are protected in Nigeria under Schedule 1 of Decree No. 11 (1985): Control of International Trade in Endangered Wild Fauna and Flora[4,7].

As the market value of wildlife has escalated with increasing demand and decreasing supply, there has been a marked shift in hunting motives from primarily for subsistence to purely for trade purposes. The resultant over-hunting has exposed several species, most especially the mammals to increased the risk. Pangolin is one of the mammalian species most affected [21]. This vulnerability of mammals to high incidence of utilisation reflected in some previous studies. Fifty-eight percent of the animals documented by [10] were mammals while among the more than five taxa recorded in [19], mammals represented the second largest utilised group.

The present study was designed primarily to compare the trend of utilisation for this species between different locations and amongst different peoples in the country. While the data in Soewu and Ayodele (2009) [7] dwelt primarily on the volume traded, the present study focused on the actual rate of utilisation as revealed by the traditional Yorubic medical practitioners. This rate of utilisation provides a useful index of the cropping pressure on populations of this animal in the wild. Also, in addition to some aspects examined in the previous study, questions were raised on complementary ingredients required for the preparations - some of which on their own may be of conservation interests - methods of preparation cum administration of the trado-medical remedies. It also identified possible wild animal substitutes with their parts that could be used successfully in place of pangolin.

## Methodology

**Figure 1:**
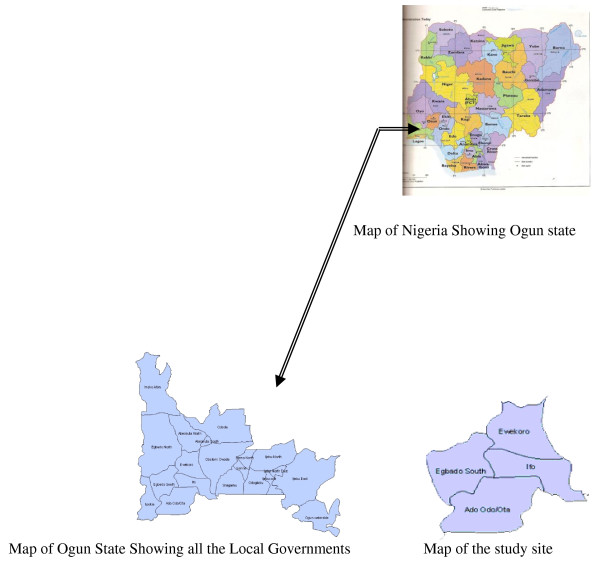
**The map of Nigeria and Ogun State showing study site**.

The study was conducted in Ifo, Ewekoro, Egbado South and Ado Odo/Ota Local Government areas in Ogun State, Nigeria (Figure 1) between December 2006 and March 2007. Ogun State is entirely in the tropics. Located in the Southwest zone of Nigeria with a total land area of 16,409.26 square kilometres, it is bounded on the West by the Benin Republic, on the South by Lagos State and the Atlantic Ocean, on the East by Ondo State, and on the North by Oyo and Osun States. It is situated between Latitude 6.2°N and 7.8°N and Longitude 3.0°E and 5.0°E. It has an estimated population of 3,486,683 people for the year 2005 [7].

## Survey

Stratified random-sampling technique was employed to select the respondents throughout the study. Open-ended questionnaire was used to encourage maximum discussion and optimum extraction of information. A preliminary survey was conducted in December 2006 to standardise the questionnaire, determine the time required to completely administer one questionnaire and, establish contacts with the tymps association. Ten questionnaires were administered for the preliminary survey in all the Local Government Areas included in the study. During the main survey which spanned January-March 2007, questionnaires were administered on a total of 40 traditional Yorubic-medical practitioners (tymps) i.e 10 tymps in each Local Government Area. Questionnaires were administered on the tymps by direct interview method.

The study investigated the specific parts of pangolins employed to treat various conditions; complimentary ingredients required; method of preparation cum administration; need for accompanying incantations; and substitute wildlife species (and their parts) that could replace pangolin parts without necessitating a change in other ingredients. The quantity of pangolin utilised for the period as well as observable trends in size and availability of the animal were also examined. Demographic data were also collected on the practitioners. The survey was carried out in all the four local government areas simultaneously.

Three types of pangolins exist in West-Africa: the giant pangolin (*Manis gigantea*), the tree pangolins (*Manis tricuspis*) and the ground pangolin (*Manis temminckii*) [22].

Taxonomy, description, distribution and habitat, behavior, diet and reproduction for this animal is as presented in [7]. Although majority of its uses attributed to its scales and carcass, pangolins are also used in food as a supplementary protein source and as adornments [4,7,23].

## Results and Discussion

**Figure 2:**
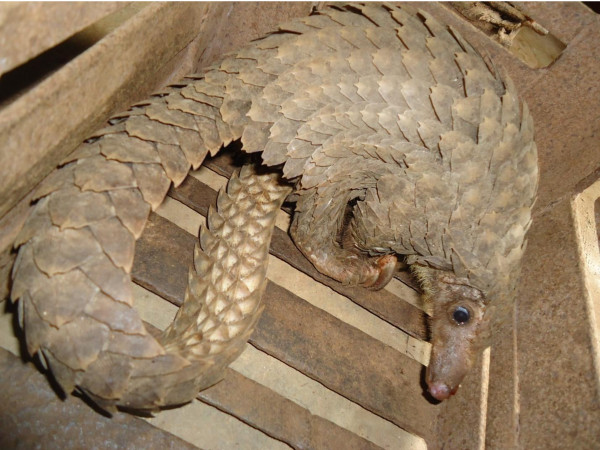
**Live pangolin in a market stall**.

Eighty percent of respondents were aged between 46 and 75 years, 5.0 percent were above 75 years of age, only 15 percent were 36-45 years old while none of them was younger than 36 years as shown in table 1. Mean age for respondents was 58.5 years (x = 58.5, n = 40). Gender distribution of respondents showed that 90% were males (table 1). Traditional medical practices dwells a lot on on-the-job experience. Table 2 shows that sixty-five percent of respondents had spent 26 years or more on the healing practice, with mean duration in practice being 30.75 years (x = 30.75, n = 40). Regarding the level of education of the respondents 22.5 percent had no formal education, 7.5 percent had exclusive quoranic education, and 55 percent had just primary education while only 5 percent had post secondary education as shown in table 3. Some respondents however combined quoranic with western education. As revealed in table 4 which depicts the source of the animal, 72.5 percent purchased pangolin from retail traders in the various markets, 25 percent bought from hunters while less than 3 percent cropped the animal directly from the wild. All respondents opined that all the pangolins they utilised, notwithstanding the point of procurement, came ultimately from populations in the wild. This agrees with the findings of Marshall (1998) [13] which reported that all plants and animals traded for traditional medicinal practices in South-Africa came from the wild. Also, [7] documented that all pangolins traded for and utilised in traditional medicinal practices by the Ijebus in Nigeria came from populations in the wild. Ten percent of respondents procured the animals by chance/on encounter, above 47% engaged in prepayment for pangolins; while about 43% claimed they contracted hunters or poachers for deliberate searches for the animals as shown in table 5. Incidence of contract hunting is slightly higher in this study with 42.5 percent of respondents involved than in [7] which recorded only 14 percent. Contract hunting is employed whenever there is an urgent need for the animal, whole or parts, but which is not readily available in nearby markets. High incentives often attached to contract hunting encourage poachers to push deeper into the natural habitat for this animal. In addition to depleting the population of the animal, this practice may inadvertently promote destruction of the habitat during desperate searches, thereby exposing individuals remaining in the population to further risks [7]. Table 6 presented the trends observed by respondents as regards the availability and size of pangolin. More than 92 percent believed that the availability of the animal in terms of its abundance is steadily decreasing with 5 percent yet to notice any difference in this trend. As regards the size, well above 97 percent claimed to have observed a continuous decline over time in the size of pangolins they use. The quantity of pangolin utilised by the respondents over a period of one month is shown in table 7. On the overall, 40 practitioners utilised 64 whole pangolins (Figure 2), giving an average of 1.6 animals per tymp per month.

**Table 1:** Age and gender distribution of respondents

Age (in years)	Frequency	Percent
36-45	6	15.0
46-55	10	25.0
56-65	12	30.0
66-75	10	25.0
76-85	2	5.0
Total	40	100.0
Gender		
Male Female 4	36	90.0 10.0

**Table 2:** Duration in Practice

Duration (in years)	Frequency	Percent
6-15	5	12.5
16-25	9	22.5
26-35	10	25.0
36-45	12	30.0
46-55	4	10.0
Total	40	100.0

**Table 3:** Level of Education of Respondents

Level	Frequency	Percent
None	9	22.5
Quoranic	3	7.5
Primary	22	55.0
Secondary	4	10.0
Post-secondary	2	2.0
Total	40	100.0

**Table 4:** Source of Animal

Source	Frequency	Percent
Direct cropping	1	2.5
Buy from hunters	10	25.0
Buy from retail dealers	29	72.5
Total	40	100.0

**Table 5:** Mode of Procurement

Mode	Frequency	Percent
By chance	4	10.0
Prepayment	19	47.5
Contract hunting	17	42.5
Total	40	100

**Table 6:** Trends in availability and size of pangolin

Abundance		
**Trend**	**Frequency**	**Percent**

Increasing	1	2.5
Decreasing	37	92.5
No difference	2	5.0
Total	40	100.0

Size		

Trend	Frequency	Percent
Increasing	0	0
Decreasing	39	97.5
No difference	1	2.5
Total	40	100.0

**Table 7:** Quantity of Pangolin used per month

Average number of pangolin used	Frequency	Percent
1	23	57.5
2	12	30.0
3	3	7.5
4	2	5.0
Total	40	100.0

Pangolin was used in treating a total of 47 conditions among the Aworis (table 8). The bone (from any part of the body), vertebral bones, eye, limbs and complete female reproductive organ were each used to treat a condition - rheumatism, stroke, kleptomania, fortune rouser and ejection of placenta respectively. Whole animal, flesh (in parts) and the complete thorax of pangolin were each employed in the treatment of 4 different conditions. A whole animal is required in the preparation of fortune rousers, immune boosters and rituals performed during the foundation laying stage of new buildings. A few pieces of pangolin carcass were used in divination and charms for good luck, protection and safety. Thorax was used to treat convulsions, unconsciousness, menstrual pains and in preventing or wading off rain. Whole internal organs of pangolin were used in preparing antidotes for food and sexual poisons while the full length tail was used to treat kleptomania and prepare charms to boost farm productivity. Pangolin head was used in the treatment of mental illness, kleptomania and in preparing good luck charms. The scale of pangolin was used to take care of 15 conditions which include stomach ulcers, venereal diseases, stroke, back pains, rheumatism, mental illness and as antibiotics. Scales were also used in preparation of medicine for safe parturition, increased productivity on the farm, wading off witches, protection and safety. Some situations required a combination of pangolin parts. Head and tip of the tail were combined in preparation of medicine for breakthrough in business, whole internal organs and complete limbs were used for money rituals while the head and scale were employed in medicine that confer protection against negative forces and their influences. The Aworis utilised pangolin extensively in traditional medicinal practices. Situations accommodated include those that can be treated by orthodox medicine as well as some that are out of range for orthodox medicine.

**Table 8:** Part of pangolins used, conditions treated, complementary ingredients, method of preparation cum administration, and substitute animal

S/No	Parts used	Conditions Treated	Other Ingredients	Method of Preparation	Method of Administration	Substitute Animal	Animal Part
1	Bone	Rheumatism	A variety of leaves, "iyere"	Made into concoction	To be taken once daily	None	
2	Eye	Kleptomania	Two whole pods of *Aframomum melegueta*	Roasted, grinded into powdery form	Used to make incision on the lower eyelids, left eye of pangolin for making incision on the left eyelid, ditto for the right eye	Python	Eye
3	Female reproductive organ	Ejection of placenta	*A melegueta *seeds, a variety of leaves	All ingredients are grinded into powdery form	To be taken with pap	Female tortoise	Whole animal
4	Flesh	To confer abilities for divination	*A melegueta *seeds, leaf of "ori okan"	Made into concoction	To be ingested at once	Wall gecko, Parrot	Whole Flesh
5	Flesh	Good luck	A variety of leaves, *A melegueta *seeds, whole hare	Made into concoction	To be consumed all at once	None	
6	Flesh	Protection	A variety of leaves, *A melegueta *seeds	Made into concoction	To be consumed all at once	Whole tortoise	Flesh
7	Flesh	Safety	'Oriji' leaves	Made into concoction	To be consumed all at once	None	
8	Head	Good luck	"Ire, aje, sawerepepe" leaves, black soap	Roasted, grinded into powdery form, mixed with soap	Used to bath every morning	None	
9	Head	Good luck	*Ficus exasperata *leaves, *3 pieces of Cortiles colocynthis*, 3 *A melegueta pods*	All ingredients roasted, grinded into powdery form.	To be taken with solidified pap i.e "eko" once every Thursday	None	
10	Head	Kleptomania	Left arm of chameleon	Roasted, grinded into powdery form	Used to make incision on the lower eyelids	None	
11	Head	Kleptomania	Human faeces (of the client)	Roasted, grinded into powdery form	Used to make incision on lower eyelids and ingested with water	Python	Head
12	Head	Kleptomania	"Alupaida" and "Ewon pabida" leaves	Roasted, grinded into powdery form	Used to make incision on lower eyelids and ingested with pap	Crab	Whole
13	Head	Mental Illness	A variety of leaves and roots, a chunk of he-goat skull	All ingredients grinded together	To be consumed twice daily	Gorilla	Head
14	Head + tip of the tail	Breakthrough in business	A variety of leaves, soap	Ingredients grinded, mixed together with soap	To be used to bath once in a week	None	
15	Internal organs	Antidote for food poison	Variety of leaves	Made into liquid mixture	To be drunk twice daily	None	
16	Internal organs	Antidote for food poison	Urine of a virgin male/female	Internal organs soaked in urine for 7 days	To be drunk and used to rub the body	Cobra	Internal organs
17	Internal organs	To treat sexual poison 'magun'	"Awogbaarun" roots	Made into decoction	To be drunk immediately after attack	None	
18	Limbs	Fortune rouser	7 *A melegueta *seeds	All the limbs of a pangolin are grinded with *A melegueta *seeds and buried in a dump site for 7 days, afterwards made into concoction	To be consumed all at once	None	
19	Limbs+ Internal organs	Money rituals	16 pieces of *Ficus exasperata* leaves,3 whole *A melegueta *pods	Leaves and A melegueta seeds seeds grinded, used to cook pangolin parts into concoction	To be consumed all at once	None	
20	Scale	Good luck	*A melegueta *seeds, shaft of melon seeds	Roasted, grinded into powdery form	To be taken with pap	None	
21	Scale	Back pain	*A melegueta *seeds, shea butter, some leaves, "kafura pelebe"	Other ingredients grinded and mixed with shea butter	To be used to rub the back twice daily	None	
22	Scale	Healing of wounds/cuts	*A melegueta *seeds	Roasted, grinded into powdery form	Powder sprinkled on the cuts/wounds and covered with a piece of cloth	None	
23	Scale	High productivity on the farm	Goat fat, porcupine spine	All ingredients roasted and grinded.	To be sprinkled on the farm	None	
24	Scale	High productivity on the farm	Whole tortoise *Mucuna pruriens *seeds, a variety of leaves	All ingredients roasted and grinded	To be sprinkled on the farm	None	
25	Scale	Kleptomania	*A melegueta *seeds	Roasted, grinded into powdery form	To be taken with pap	None	
26	Scale	Mental illness	Mainly incantations	Scales grinded for use (7 pieces for females and 9 for male)	A single dose to be ingested once daily with incantations for 16 days	None	
27	Scale	Rheumatism	Shea butter, a variety of leaves	Other ingredients grinded and mixed with shea butter cream	To be used to rub affected parts	Python	Spinal cord
28	Scale	Stomach ulcer	Dead earthworm found on the road	Grinded into powdery form	To be taken with pap	None	
29	Scale	Stroke	*A melegueta *seeds, a variety of leaves	All ingredients grinded, made into a decoction	To be drunk once daily	None	
30	Scale	Venereal diseases	A variety of leaves	Roasted, grinded into powdery form	To be taken with hot water	None	
31	Scale	Wading off witches	Incense burner, "Eepo obo", "eerun", "imi ojo"	All ingredients grinded and poured in incense burner	Burnt as incense indoors	None	
32	Scale (whole)	Safe delivery	*A melegueta *seeds and a variety of leaves	Roasted and grinded into powdery form	To be taken with pap	Porcupine	Spine (whole)
33	Scale	Good luck	Bitter leaf, *A melegueta *seeds, "Oriji" leaves, local soap	All ingredients roasted, grinded, mixed with soap	To be used to bath every 3 days	Male lizard	Whole animal
34	Scale	Aphrodisiacs/male potency	*A melegueta *seeds, a variety of leaves	Roasted, grinded into powdery form	To be taken with pap continously	None	
35	Scale (whole), head	Protection	*A melegueta *seeds, porcupine spine	Roasted, grinded into powdery form	To be ingested with pap	Cobra	Head/ whole skin
36	Scale	Antibiotics	Hare carcass, gun powder, "iyere", "Kafura pelebe"	All ingredients grinded	To be taken with pap	None	
37	Thorax	Convulsion	A juvenile dog, *A melegueta *seeds, a variety of leaves	Made into concoction	To be consumed as soup	None	
38	Thorax	Menstrual pain	A whole crab, shrew,*Citrullus colocynthis*, 'iru' melon, 'ogiri'	All ingredient cooked into concoction	10 oz to be drank 3 times daily	None	
39	Thorax	Unconsciousness	"Igi aaka" roots	Grinded into powdery form	To be swallowed with water	None	
40	Thorax	Wading off/preventing rain	A whole *A melegueta *pod, a variety of leaves, a padlock	All ingredients, grinded, packed in a piece of rag, tied to the padlock	Incantation is recited on the padlock, it is nailed to a tree	None	
41	Vertebral bones	Stroke	Riverside banana, "ifon", roots "eru alamo"	Cooked into concoction	To be taken 3 times daily	None	
42	Whole animal	Building rituals	Palm oil, Salt, "Iyere", "Olugelegele" leaves	Blood is placed in a new plate, and the whole flesh is divided into 16 pieces	Blood poured on the floor, and flesh eaten as concoction	None	
43	Whole animal	Good fortune	Sponge used to bath a human corpse, a whole pod of *A melegueta*, "owo ara tangiri" soap	All ingredients grinded, mixed with soap, poured in a white container	To be used to bath on Sunday, Monday, Tuesday	None	
44	Whole animal	Prosperity	*A melegueta *seeds, black soap	All ingredients grinded and mixed with black soap	To be used to bath regularly	None	
45	Whole animal	Wading off/curing bad illness & sickness	*A melegueta *seeds, a variety of leaves, black soap	Roasted, grinded into powdery form, mixed with soap	To be used to bath regularly	None	
46	Whole tail	High productivity on the farm	Porcupine spine, "jiwini", leaves *Mucuna pruriens *leaves "ekuru"	All ingredients grinded into powder	To be sprinkled on the farm	None	
47	Whole tail	Kleptomania	A variety of leaves	Grinded into powder	Used to make incision on the wrists and taken with pap	None	

Diversity of conditions treated and parts employed in this study are similar in some cases to findings of some previous studies on trado-medicinal practices among other peoples. In previous single species studies, [24] reported that fat and egg of *Podocnemis expansa *were used to treat 16 different diseases while [7] reported the use of pangolin to treat 42 conditions amongst the Ijebus.

Traditional Yorubic medicine among the Ijebus used pangolin scales to treat stomach ulcers, venereal diseases, stroke, mental illness, to wade off witchcraft and to prepare traditional antibiotics as also recorded in this study. Other areas of similarities in uses between the Ijebus and the Aworis include the use of eyes to treat kleptomania, use of bones to treat rheumatism and stroke and, the utilisation of pangolin head for good luck charms. Some points of divergence in utilisation pattern between Ijebus and Aworis were recorded as there were some similar situations that required different parts of the animal. While the Ijebus used pangolin scale to prepare antidotes for sexual and food poisons, the Aworis in this study utilised whole internal organs of pangolins for such preparations. The Ijebus required the head to treat convulsions, but the Aworis would utilise the thorax for same purpose. Also the Ijebus will require a whole animal to prepare charms for breakthrough in business whereas the Aworis will employ the head and tip of tail for the same purpose.

The head of pangolin was used along with some other ingredients to treat kleptomania in this study. This agreed with [3] which stated that the head of white-bellied pangolin, *Manis tricuspis*, same species encountered in this study with the eyes intact was used in curing/treating kleptomania. The whole animal was employed to cure/wade off bad illness and sickness while [1] reported that the scale was utilised to cure skin diseases. The thorax of pangolin was used to treat menstrual pain in this study but [25] reported that pangolin scales were believed to help regulate menstruation and stabilise breast milk secretion. Also the thorax of pangolin was used to wade off/prevent rain as against [7] which reported that the scales and blood were used for rain making and to protect against bad omen and prepare amulets against gun shots. Some group of people in East India utilised the scales for rheumatism and labour pain. The Chinese used the scales for preparations to neutralise witchcraft and evil spirits and to cure sores [7]. Scales were also employed in the present study to treat open cuts (sores) in the body.

The scale has the highest fidelity level in this study i.e. it is the most frequently utilised part in traditional medicinal preparations - a total 15 conditions were treated using the scales of pangolin. In addition to documenting pangolin scales as having the highest fidelity level, [25] also reported that South Korea imported a total of 29, 621 kg of pangolin scales valued at USD 471,000 from China, Vietnam, Indonesia and Singapore between 1993 and 1994 for use in the Traditional Korean medicine (TKM). In the present study, the scale was used for ejection of placenta in women after delivery but [3] reported that a whole female pangolin was required for the extrusion of placenta after parturition in women. The scales were also used to wade off witches among the Aworis and this agrees with [25] which reported that the scales were thought to neutralise witchcraft and evil spirits. It is worthy to emphasise here that the scales cannot by any means be extracted without killing the animal.

The utilisation of pangolin in treating the various conditions identified was found to be guided by a number of factors suggesting a unique co-evolution between medical, social and ecological systems. This agrees with some of the findings in Soewu (2008) [15]. The bioactive ingredients in some parts of this animal were responsible for some of their uses. Use of scales to treat wounds, stomach ulcer and venereal diseases is premised on anti-microbial potencies of preparations from the scale. Behavioral and ecological tendencies observed in the animal provided another guiding factor. The treatment of kleptomania with the eyes result from observed shyness in the animal. Some mythological conceptions about pangolin also influenced the use of some parts of the animal to treat some situations. Employment of female reproductive organs in preparations for safe delivery and ejection of placenta during childbirth arose from reasons that are apparently psychological or mystical. Preparations of medicines used as fortune rousers and to boost farm productivity were also found to dwell more on perceived mystical properties of this species.

An average of 1.6 pangolins utilised per tymp in a month is beyond the sustainable level for this species which requires at least two years attaining sexual maturity, gestation period of 150 days and has a litter size of just one. Although pangolin is presently not directly under the threat of extinction, it is listed in appendix II of CITES and schedule 1 the Nigeria's Endangered Species (Control of International Trade and Traffic) Decree No 11 1985. This requires that trade in this species must be regulated in order to avoid unsustainable utilisation of the species which may further worsen its conservation status. Findings during this study show that it is either the respondents have a total lack of awareness of the existence and implications of *Decree No 11*, or they know that the law is not enforced. With no record of successful captive breeding or domestication yet, especially in this part of the world, the only source of this animal is from populations in the wild that are already fast declining due to over-exploitation for medicinal uses [7]. This means a steady demand for a natural resource whose population size in the wild has not been established to be either adequate to cope with present demand or expanding appropriately to cope with likely increased demand in future.

Regarding the use of substitute animal, only 13 out of the 47 situations encountered during this study (27.7 percent) would readily accommodate another animal in place of pangolin. However, some of the animals identified as possible substitutes for pangolin are worse off regarding their conservation status as indicated in the various listings. Gorilla is actually listed on schedules I and 1 of CITES and Nigerian Decree No 11 respectively. Parrot and python occupy the same position as pangolin on these lists. Other substitute animals are not yet listed. This stresses the need for restraint when advocating the use of substitute animals in traditional medicines to avoid creating more conservation problems while trying to solve one.

The practitioners were often reluctant to disclose the full complement of ingredients required for some preparations. This might have stemmed from their attempt to safeguard the secrets of the healing art which is the only source of livelihood for virtually all of them and protect their heritage of several generations.

## Conclusion

The use of this animal in traditional medicine is intensive and has no consideration for either the present conservation status of this species or the sustainability of such utilisation for the animal. There is a need to educate the entire citizenry on the implications of a total loss of this specie as a result of over-exploitation for biodiversity conservation and health care delivery. Traditional Yorubic-medical practitioners should be enlightened on the status of animals used in their trado-medical preparations. Most practitioners fail to realise that if these animals go into extinction, the lives of people who solely depend on traditional medicine would be at risk and their own trade may be adversely affected. If the trend of utilisation is not addressed sustainably, the practitioners of ethnomedicine would be at a greater risk of extinction than forests and other biomes in a manner akin to the current spasm of plant and animal extinction [14]. Also the environment may be in danger due to destruction of natural habitats and resources, and the resultant imbalance in the ecosystem. While advocating that the medicinal use of animals be considered together with other anthropogenic pressures, [14] observed that rapid reduction in natural resources as a consequence of the expanded urbanisation, global warming and reduced natural habitat poses a considerable threat to the sustainability of traditional medicine. It is very important for conservation groups to create adequate awareness as regards the implications of depletion of natural resources (flora and fauna) having known medicinal values.

A field study to assess the population dynamics of this animal in the wild is urgently required. According to [24] there is a need to increase our understanding of the biology and ecology of species commonly used as remedies to better assess the impacts of harvesting pressure (for medicinal and other purposes) on their wild populations. The report [24] stressed further that the general acceptance of zootherapy calls for an assessment of the impact of this healing method on wild populations. Efforts should also be intensified on ex-situ breeding projects to raise pangolins in captivity for the consumptive uses as well as re-introducing them into the wild. This may reduce the pressure on pangolin populations in the wild. The conservation of medicinal wildlife resources will require conservation, management, awareness, regulation and research initiatives by a whole range of institutions. Having recorded the highest fidelity level in previous studies as well this survey, there is need to subject scales as well as other parts of the various species of this animal to laboratory studies to determine the bioactive ingredients in them that makes pangolin so important medicinally all over the world.

## Competing interests

The authors declare that they have no competing interests.

## Authors' contributions

DAS conceived of the study, participated in its design and coordination, review of literature and revision of the manuscript. TAA participated in data collection during the preliminary and main survey and in drafting the manuscript. Both authors read and approved the final manuscript.
